# Gene-Regulatory Logic to Induce and Maintain a Developmental Compartment

**DOI:** 10.1371/journal.pgen.1005543

**Published:** 2015-10-15

**Authors:** Marco Milán

**Affiliations:** 1 Institute for Research in Biomedicine (IRB Barcelona), The Barcelona Institute of Science and Technology, Barcelona, Spain; 2 Institució Catalana de Recerca i Estudis Avançats (ICREA), Barcelona, Spain; The University of North Carolina at Chapel Hill, UNITED STATES

Forty-four years ago, Antonio García-Bellido utilized, for the first time in biology, the clonal analysis technique to characterize the parameters of proliferation and growth of a developing organ—in this case, the highly proliferative wing primordium of *Drosophila* [1]. A couple of years later, using the same technique, García-Bellido and two of his PhD students, Ginés Morata and Pedro Ripoll, reported the existence of adjacent cell populations that do not mix, which he called compartments [2]. Developmental compartmentalization of growing organs was subsequently demonstrated in all ectodermal derivatives of the fruit fly, as well as in vertebrate limbs and in the central nervous system. One of the most remarkable features of compartments is that their boundaries also act as organizing centers. Compartments are defined by the restricted expression and activity of the so-called selector genes. The homeodomain-encoding gene *engrailed* was rapidly identified as the one specifying the posterior compartment of all ectodermal derivatives of the fruit fly [3,4]. It was not until the early 1990s that the LIM-homeodomain-encoding gene *apterous* (*ap*) was shown to specify the dorsal compartment in the *Drosophila* wing [5]. These selector genes share three distinct activities: specification of compartment identity, localization of an organizing center at the compartment boundary, and establishment of the lineage restriction border. The *ap* selector gene rapidly became a paradigm in the identification of the signaling molecules, cell adhesion proteins, and transcription factors that mediate the construction of compartments and organizing centers [6–9]. However, how *ap* expression is maintained in order to fulfill its functions in all the cells of the dorsal compartment during the five days of growth and proliferation has remained largely unknown. In this issue of *PLOS Genetics*, Carlos Estella and colleagues [10] unravel the molecular mechanisms underlying the initiation and maintenance of *ap* expression in the *Drosophila* wing primordium. A combination of enhancers, autoregulatory and feed-forward mechanisms, and epigenetics appears to be at play.

Perhaps the best way of identifying the noncoding regions involved in the regulation of expression of a gene of interest is through classical genetics and phenotypic characterization. Indeed, this is the approach taken by Estella and colleagues in their analysis of the *ap* locus. A series of endogenous deletions were performed to identify the regulatory sequences required to drive *ap* expression and give rise to normal-looking adult wings. By doing so, the authors functionally identified a proximal region harboring a Polycomb Response Element (PRE) and a distal fragment containing a previously identified enhancer, whose deletions severely compromised *ap* expression and wing development. The authors next undertook a systematic in situ dissection of the *ap cis*-regulatory domain by bringing back sub-fragments of a previously deleted region and analyzing their capacity to rescue the wing phenotype. When combined, the PRE and two enhancers located in a distal fragment were sufficient to rescue both *ap* expression and wing formation.

So far so good, but what is the regulatory logic that combines the input of two distinct enhancers and a PRE to induce the expression and maintenance of *ap* expression in all dorsal cells throughout development? The authors again approached this issue in a clever manner. They constructed a battery of *lacZ* reporter lines to decipher how these enhancers were expressed in the wing, to identify the transcription factor involved in their regulation, and to analyze the contribution of the PRE to this process. As expected, the two functional enhancers located in a distal fragment and identified by the in situ rescue experiment drove expression of the *lacZ* reporter in dorsal cells of the wing primordium. The isolated *ap-E* enhancer is initially expressed in all dorsal cells and responds to the early activity of the Epidermal Growth Factor (EGF)-Receptor ligand Vein, as does the whole *ap* locus [11]. Interestingly, its initial expression coincides in time with the onset of *ap* expression and the initiation of the organizing activities of the compartment boundary through the activation of the Notch receptor. Later in development, expression of the *ap-E* enhancer fades away from the dorsal compartment of the wing primordium, and the *ap-DV* enhancer starts to be expressed in exactly the same region as a consequence of the activity of the Apterous and Vestigial proteins. It is interesting to note that Vestigial is a target of Notch [12]. Thus, both an Apterous-dependent autoregulatory mechanism and a feed-forward mechanism, through the activity of Notch and Vestigial, drive the expression of the *ap-DV* enhancer. The authors observed that these two enhancers were not able—either alone or in combination—to completely reproduce the *ap* expression pattern. Thus, additional elements appear to be at work. Following the logic of the in situ rescue experiments, the combination of the *ap-E* and *ap-DV* enhancers, together with the *ap-PRE*, gave rise to robust expression of the *lacZ* reporter in all dorsal cells throughout wing development. Interestingly, the ability of the *ap-PRE* to drive robust expression was shown to rely on the activity of the Trithorax group of proteins.

**Fig 1:**
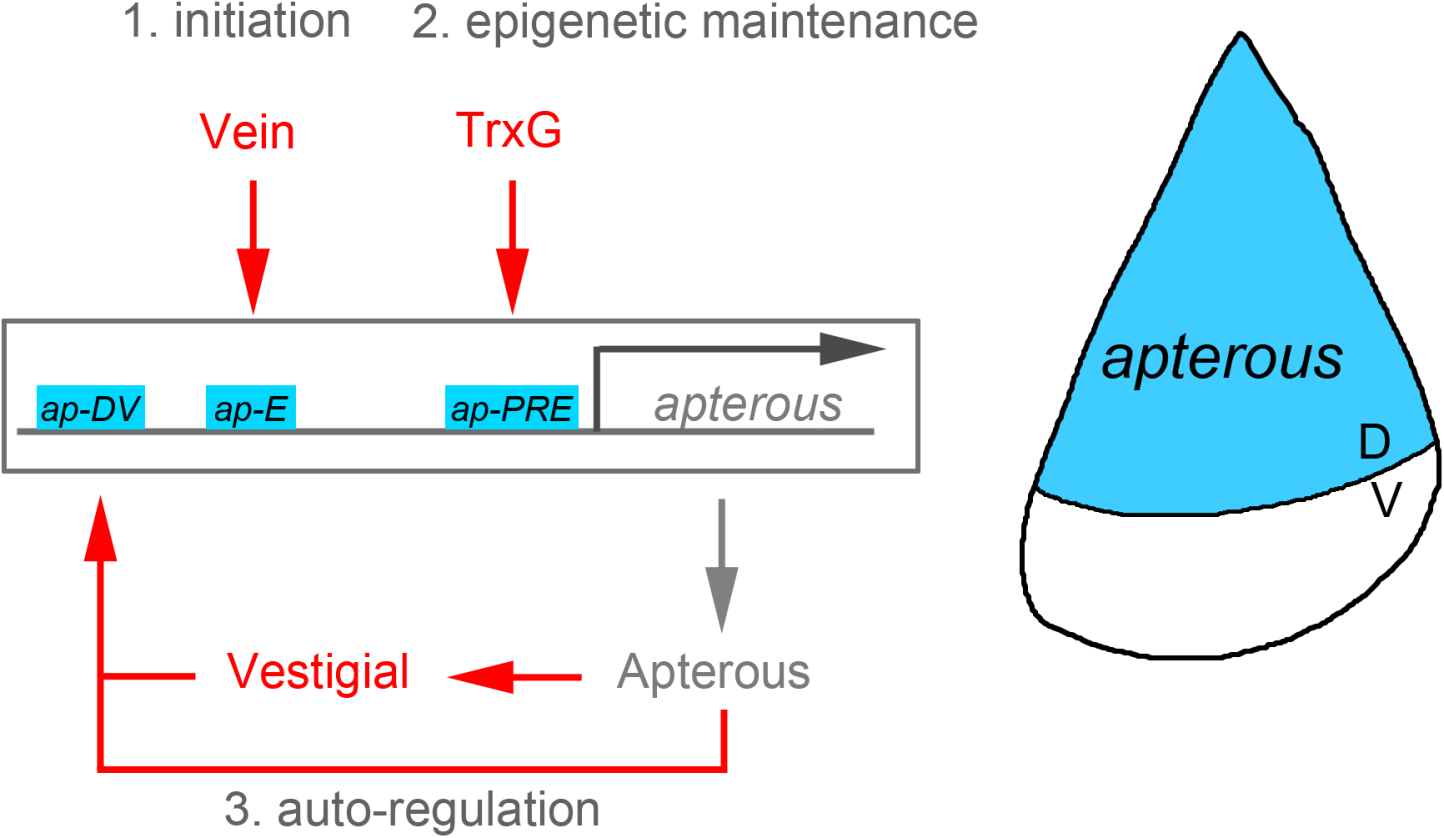
Illustration of the *apterous* locus with its regulatory regions involved in the control of its expression in the *Drosophila* wing. Whereas the onset of *apterous* expression in the dorsal compartment of the early wing primordium relies on the activity of the EGF receptor ligand Vein, the maintenance of its expression during subsequent developmental stages depends on both autoregulatory and epigenetic mechanisms.

Taken together, the results from Estella and colleagues reveal a three-step molecular mechanism to initiate and maintain *ap* expression in all dorsal cells throughout development (Fig 1). Whereas the onset of *ap* expression relies on the restricted expression of the EGF receptor ligand Vein and on the activity of the EGF-Receptor responsive transcription factor Pointed-P1 in the presumptive dorsal compartment, the maintenance of *ap* expression during the subsequent stages of wing development is led by an Apterous-dependent autoregulatory loop, a Vestigial-mediated feed-forward mechanism, and the activity of the PRE. This initiation and maintenance mechanism has remarkable commonalities with the logic followed by other developmental genes whose expression has to be precisely initiated but also robustly maintained throughout the dramatic increase in tissue size and cell number that occurs in the primordia of the adult fly [13,14]. Forty-two years after the discovery of compartments, the *Drosophila* wing is still unraveling common principles of development.
